# Exploring the Gap Between Needs and Practice in Facilitating Breastfeeding Within the Neonatal Intensive Care Setting: An Italian Survey on Organizational Factors

**DOI:** 10.3389/fped.2019.00276

**Published:** 2019-07-18

**Authors:** Elisabetta Tambani, Maria Lorella Giannì, Elena Nicoletta Bezze, Patrizio Sannino, Gabriele Sorrentino, Laura Plevani, Daniela Morniroli, Fabio Mosca

**Affiliations:** ^1^Fondazione IRCCS Cà Granda Ospedale Maggiore Policlinico, NICU, Milan, Italy; ^2^Department of Clinical Sciences and Community Health, University of Milan, Milan, Italy; ^3^Fondazione IRCCS Cà Granda Ospedale Maggiore Policlinico, Direzione Professioni Sanitarie, Milan, Italy

**Keywords:** Baby Friendly Hospital Initiative, breastfeeding, NICU, organizational context, preterm infants

## Abstract

**Introduction:** The system-level factors of the neonatal intensive care unit work environment contribute to breastfeeding promotion in the preterm population. The aim of this study was to investigate the operative policies related to breastfeeding support in a sample of Italian Neonatal Intensive Care Units.

**Materials and Methods:** A multicenter cross-sectional survey was conducted, including a sample of 17 head nurses. The items of the questionnaire investigated the following areas: breastfeeding policies, staff education, family centered care, and breastfeeding promotion and support both in the neonatal intensive care units and after discharge.

**Results:** Written breastfeeding policies were available for staff in all the neonatal intensive care units, most commonly addressing procedures related to skin-to-skin contact, human milk expression, and preterm infant breastfeeding. Most of the neonatal intensive care units correctly advised the mothers to initiate milk expression within 6 h from delivery and to pump milk at least 6 times/days. Breastfeeding training for the nursing staff was planned in the majority of the neonatal intensive care units although according to different schedules. With regard to the family centered care implementation, time restrictions were present in seven neonatal intensive care units, mostly occurring during the night shift, and the morning hours concomitantly with medical rounds. Moreover, in the majority of the investigated neonatal intensive care units, the parents were asked to leave the ward when their infant underwent a major invasive procedure or during the nurse/physician shift change report. With regard to breastfeeding promotion and support, eight neonatal care units had a multidisciplinary team with several health care professionals and 10 provided information about community-based support services. Most of the units assessed breastfeeding after discharge.

**Conclusion:** Based on the present findings, enrolled Neonatal Intensive Care Units appear to provide breastfeeding-supportive environments with special regard to breastfeeding policies, milk expression practices, interprofessional collaboration, and continuity of care. Health care professionals should exert efforts to ensure continuous and updated breastfeeding staff education and promote parent-infant closeness and family centered care.

## Introduction

Breastfeeding provides health benefits to both the infant and the mother (1) that are even more pronounced for preterm infants (2–4). Human milk feeding is associated with better enteral feeding tolerance; improved neurodevelopmental outcomes; decreased rates of late-onset sepsis, necrotizing enterocolitis, chronic lung disease and retinopathy of prematurity; and fewer rehospitalizations at 18 and 30 months of age (5). Noteworthy, these beneficial effects are dose dependent (6).

The birth and hospitalization of a preterm infant creates unique challenges to the breastfeeding process due to several barriers, such as infant health status, parent/infant separation, staff workloads, and the physical environment of the Neonatal Intensive Care Unit (NICU) (7–9). This complex scenario is reflected by the lower rates and durations of breastfeeding in the preterm population than recommended (10).

The implementation of the Baby-Friendly Hospital Initiative (BFHI), which adapted its 10 steps to the NICU context, has been advocated to promote mother's milk feeding in this population (11). Within this context, the importance of promoting family-centered care during hospital stay has been further underlined (12). A recent survey, including a high number of participating wards, has brought to light the international readiness to expand Neo-BFHI designation (13).

The system-level factors of the NICU work environment have been shown to contribute to the success of the Baby-Friendly Hospital Initiative adoption (14). However, the role of hospital breastfeeding policies, health care staff disposition, and NICU organization as contributing factors to breastfeeding success has not been fully explored (15).

The aim of the present study was to investigate the organizational factors that are related to breastfeeding support in a sample of Italian NICUs.

## Materials and Methods

### Design and Setting

A multicenter cross-sectional survey was conducted, including a convenience sample of 17 head nurses, 16 working in northern Italy NICUs and one in southern Italy. Among the contacted NICUs, only one refused to participate. Data was collected from December 2016 to February 2017. The Ethics Committee of the Fondazione Istituto di Ricovero e Cura a Carattere Scientifico Cà Granda Ospedale Maggiore Policlinico approved the study and written informed consent was obtained by all participants.

### Data Collection

The investigator in charge of the study contacted via e-mail each head nurse to arrange an appointment by phone. The head nurses were then administered a structured phone interview lasting ~15 min.

### Measurement

The tool used was a modified and shortened version of the Breastfeeding Support Tool (7). A dedicated group of nurses, experts in breastfeeding in preterm infants, including an International Board Certified Lactation Consultant (IBCLC), discussed the changes of the Breastfeeding Support tool to be implemented. Specifically, 10 items from the original questionnaire were retained and four new items were included, aiming at investigating thoroughly the organizational context of the enrolled NICUs, with particular regard to the Neo-BFHI implementation.

The modified questionnaire was translated into Italian through the back-translation method and submitted to a group of head nurses to clarify any doubts regarding the comprehension of the items (Table 1).

**Table 1:** Questionnaire items.

1.	Do NICU nurses receive a breastfeeding training? If the answer is yes, please specify: Is the breastfeeding training offered only to new employees? □Yes □No Does it occur: □every year □every 2 years □every 5 years □no specific interval
2.	Do NICU nurses receive a specific training on preterm infant breastfeeding? □Yes □No
3.	What is the percentage of trained nurses? □80% receive the 20-h OMS/UNICEF course □at least 40–79% receive the 20-h OMS/UNICEF course □at least 80% receive another specific training on breastfeeding
4.	Is the NICU accessible to parents 24 h a day? □Yes □No
5.	In which occasions can the parents stay next to their infant? □medical examinations, □major invasive procedures □minimal invasive procedures □specialist examinations □nurse shift change reports □night shifts
6.	Are written breastfeeding policies available in your NICU? □Yes □No If Yes, please indicate the ones that are available: □prenatal information □donor milk, □breastfeeding of preterm infants □milk expression □the use of nipple shields □skin-to-skin contact □the use of a pacifier
7.	How many hours after delivery is it recommended to start breast expression? □ < = 6 □ < = 12 □ < = 24 < = □48 □> 48 □no recommendation
8.	How many times per day is breast expression recommended? □0–3 □4–5 □6–8 □>8
9.	What written information are given to parents? □preterm infant breastfeeding □milk expression □nipple shield use □skin-to-skin contact □pacifier use
10.	Is a peer support group available in the NICU? □Yes □No
11.	Is an IBCLC available in the NICU? □Yes □No
12.	Is a multidisciplinary team available in the NICU? □Yes □No
13.	Does the NICU staff provide information about community-based breastfeeding support services? □Yes □No
14.	Does the NICU staff assess breastfeeding even after discharge? □Yes □No

Items 1, 3, and 5–9 comprised multiple choice questions. With regard to item 1, if the answer was yes, the head nurses were invited to specify whether the training was only for new employees and if it occurred every year, every 2 years, every 5 years, or other. The item 3 answers were categorized as follows: at least 80% received the 20-h OMS/UNICEF course, at least 40–79% received the 20-h OMS/UNICEF course and at least 80% received another specific training on breastfeeding.

Items 2, 4, and 10–14 comprised dichotomic questions (yes or no).

For data presentation the items of the questionnaire were grouped according to some of the areas contained in the ten steps of the Neo-BFHI. These areas were as follows: group 1 (items 6–9) focused on breastfeeding policies, group 2 (items 1–3) focused on staff education, group 3 (items 4–5) focused on implementing family centered care, and group 4 (items 10–14) focused on breastfeeding promotion and support both in the NICU and after discharge.

NICUs' level, annual admissions in the previous year to each enrolled NICU, the eventual achievement of the Baby Friendly Hospital Initiative designation, the respondents' professional profiles (i.e., nurse, pediatric nurse, or both) and experience were further collected.

### Data Analysis

Data are expressed as the means ± standard deviation (SD) or the percentages.

Comparison among NICUs, categorized into two groups according to the median number of infants admitted in the previous year, were performed using chi-square test.

## Results

The basic characteristics of the respondents and of the enrolled NICUs are shown in Table 2.

**Table 2:** Basic characteristics of the enrolled NICUs and of the respondents.

**NICUs' characteristics**	
Level 3 (*n*)	14
Level 2 (*n*)	3
Baby Friendly hospital designation (*n*)	none
Number of annual admissions (mean ± SD)	373 ± 168
**Head nurses' professional profile/experience**	
Nurses (*n*)	12
Pediatric nurse (*n*)	4
Nurse and pediatric nurse (*n*)	1
Years of professional experience (mean ± SD)	9.7 ± 7.8

The median number of infants admitted to the NICUs, whose head nurses participated to the survey, was 348 (range 124–756). A total of eight NICUs admitted ≤348 infants, and nine NICUs admitted >348 infants.

No NICU obtained a Baby Friendly hospital designation; however, five NICUs had initiated the training to achieve it.

### Questionnaire Answers Subtopics

#### Group 1: Breastfeeding Policies

Written breastfeeding policies were available for staff in all the NICUs, most commonly addressing policies related to skin-to-skin contact, human milk expression, and preterm infant breastfeeding (Table 3).

**Table 3:** Written breastfeeding policies available for staff and written information available for parents.

	***N* (%)**
**NICUs with written breastfeeding policies**	17 (100)
**Type of breastfeeding policies available**	
Skin to skin contact	16 (94)
Milk pumping	14 (82)
Breastfeeding of preterm infant	13 (76)
Use of donor milk	11 (65)
Use of pacifier	10 (59)
Prenatal information	9 (53)
Use of nipple shield	6 (35)
**NICUs with written information available for parents**	12 (71)
**Type of written information available**	
Milk pumping	11 (65)
Skin to skin contact	10 (59)
Breastfeeding of preterm infant	7 (41)
Pacifiers	3 (18)
Use of nipple shield	2 (12)
**NICUs with no written information available for parents**	5 (29)

Most of the NICUs correctly advised the mothers to initiate milk expression within 6 h from delivery and to pump milk at least 6 times/days (Table 4).

**Table 4:** Timing of milk pumping initiation.

**Timing of milk pumping initiation**	**NICUs (n)**
Within 6 h	14
Within 12 h	1
Within 24 h	1
No recommendation	1
**Frequency of milk pumping a day**	**NICUs (n)**
More than 8 times	3
6–8 times	12
4–5 times	1
No recommendation	1

A total of 12 NICUs offered written information for parents with regard to human milk expression, skin-to-skin contact and the breastfeeding of preterm babies (Table 3). However, five NICUs did not provide any written breastfeeding information for parents.

NICUs that admitted >348 infants had adopted preterm infants breastfeeding procedures in a higher number of case than NICUs that admitted ≤348 infants (9 vs. 4, *p* = 0.029). No other differences were detected with regard to the items of this subgroup according to the number of infants admitted to NICUs.

#### Group 2: Staff Training

Data related to this area are presented in Table 5. Breastfeeding training for the nursing staff was planned in most NICUs according to different schedules and courses. Specific training on the breastfeeding of preterm infants was also planned in 41% of NICUs. No differences were detected with regard to the items of this subgroup according to the number of infants admitted to NICUs.

**Table 5:** Staff training.

	**YES (*n*)**	**NO (*n*)**
**Breastfeeding training**	12	5
**Preterm infant breastfeeding training**	7	10
**Training frequency**	**NICUs (*****n*****)**	
Every year	6	
No specific interval	4	
Every 2 years	1	
Only for new employees	1	
**Type of course**	**NICUs (*****n*****)**	
20-h OMS/UNICEF course (at least 80% of staff)	2	
20-h OMS/UNICEF course (at least 40–79% of staff)	3	
Another specific training on breastfeeding (at least 80% of staff)	5	

#### Group 3: Implementing Family Centered Care

Parents had 24-h free access in 10 NICUs, whereas time restrictions were present in seven NICUs. These restrictions mostly occurred during the night shift due to the lack of proper space and beds for the parents and during the morning hours concomitantly with medical rounds. Moreover, both in the NICUs where parents had 24-h access and in those with time restriction, parents were asked to leave the room when their infant underwent a major invasive procedure or during the nurse/physician shift change report in the majority of the investigated NICUs (14 and 12, respectively). In contrast, the parents were allowed to stay while their infant underwent a minimally invasive procedure and/or a specialist examination and/or a medical examination in 16, 15, and 11 NICUs, respectively.

NICUs that admitted > 348 infants showed a tendency to allow parents to be present during their infant's medical examination in a higher number of cases compared to NICUs that admitted ≤348 infants (8 vs. 3, *p* = 0.05). No other differences were detected with regard to the items of this subgroup according to the number of infants admitted to NICUs.

#### Group 4: Breastfeeding Promotion and Support

Data related to this area are presented in Table 6. A multidisciplinary team, including several health care professionals, such as physicians, nurses, and nutritionists, dedicated to breastfeeding, was present in 47% of the enrolled NICUs. Among these, three also had an IBCLC and one had a peer support group that is a group of mothers collaboratively working with bedside nurses and neonatologists, as a part of the NICU care team, thus contributing to breastfeeding promotion and support. Among the NICUs that did not have a multidisciplinary team, three had a peer support group, and one had both a peer support group and an IBCLC. In contrast, five NICUs did not have any of these helping figures at their disposal. Almost all NICUs assessed breastfeeding after discharge. No differences were detected with regard to the items of this subgroup according to the number of infants admitted to NICUs.

**Table 6:** Breastfeeding promotion and support.

	**YES (*n*)**	**NO (*n*)**
Is a peer support group available in the NICU?	5	12
Is an IBCLC available in the NICU?	4	13
Is a multidisciplinary team available in the NICU?	8	9
Does the NICU staff provide information about community-based breastfeeding support services?	10	7
Does the NICU staff assess breastfeeding even after discharge?	16	1

## Discussion

The present study explores the operative policies to facilitate preterm infants' breastfeeding in a sample of Italian NICUs'. On the basis of the present findings, the majority of the enrolled NICUs, irrespective of the number of admitted infants, provides a breastfeeding-supportive environment. This survey, however, allows for the identification of some specific organizational factors (i.e., staff training, provision of written information on breastfeeding preterm infants, 24 h NICU access) that needs to be further implemented in order to optimize breastfeeding support within the intensive care unit setting. The implementation of the policies that promote breastfeeding in NICUs is indeed of great importance considering the numerous health benefits of human milk feeding for preterms.

Consistently with data in literature, all of the investigated NICUs had developed breastfeeding policies, and the large majority exhibited the greatest adherence to the Neo-BFHI human milk expression recommendations (7, 11, 13, 16). Furthermore, the great part of the enrolled NICUs provided parents with written breastfeeding information related to human milk expression and skin-to-skin contact. However, it has to be taken into account that only a relatively limited number of NICUs offered written information regarding breastfeeding preterm infants whereas up to five NICUs did not provide parents with any written breastfeeding information.

Consistently with our findings, Maastrup et al. (7) conducted a survey investigating breastfeeding support in Danish NICUs and reported that 16 of 19 NICUs had written breastfeeding policies and that the written information that was most frequently provided to parents was related to human milk expression, skin-to-skin contact, and the breastfeeding of preterm infants. The strategy of providing written information about the main areas involved in preterm infant breastfeeding practice has been advocated for promoting parent education and involvement in the care of their infants (9). Accordingly, Guiding Principle 1 of the Neo-BFHI recommends that all mothers receive appropriate counseling about infant feeding and guidance for selecting options that they find acceptable and suitable for their situations and underlines the importance of having a written breastfeeding policy for the staff (17, 18).

Regarding breastfeeding training for the staff, it was planned in the majority of the NICUs that were enrolled in the present study, although there was significant variability in training frequency among the units. The importance of ensuring that staff have sufficient and updated knowledge is highlighted in the second step of the Neo-BFHI (11) and it has been further underlined in the 2018 BFHI revision (18). It is strongly recommended that all new NICU staff members are offered breastfeeding training, such as the 20-h OMS/UNICEF training, and further regular continuing education (11). Accordingly, the training of health professionals has been associated with significant improvements in initial exclusive breastfeeding rates (19).

This survey highlights some organizational areas that need to be improved in the enrolled NICUs although several breastfeeding practices are already well-implemented. Specifically, we found relatively limited parental access to the NICUs, mainly during the night shift. Furthermore, in most NICUs, parents were not allowed to be present during their infants' major invasive procedure and during the nurse/physician shift change report while in some NICUs they were asked to leave even for medical examinations or minimally invasive procedures. The importance of family-centered care, including the non-separation of infant and parents, is widely acknowledged (17, 20, 21). These findings could be partially explained by a lack of space and beds and are in line with the findings of previous studies (13, 22) that have reported limited parental access as relatively common in Southern European countries. In contrast, in Northern Europe and the UK, the majority of NICUs allow unrestricted parental access and provide facilities such as reclining chairs near the babies' cots, beds and dedicated rooms to ensure parents' presence also during night time (7, 22). Promoting the non-separation of infants and parents is crucial for empowering the parental role; family-centered care programmes in NICUs have been demonstrated to promote improved maternal confidence and higher rates of breastfeeding at 3 months after discharge (21).

The support provided by the entire health care team has been recognized as crucial to the implementation of the Neo-BFHI (11). Accordingly, half of the NICUs in the present study had a multidisciplinary team, which reflects the major role played by multidisciplinary educational interventions in the promotion of successful breastfeeding.

Remarkably, nearly all of the investigated NICUs assessed breastfeeding after discharge. This finding underlines the importance of providing continuous support to parents of preterm infant, beginning at NICU admission and continuing through the hospital stay and after hospital discharge. This concept is further stressed in the third guiding principle of the Neo-BFHI, which underlines that the health care system must ensure continuity of care (i.e., prenatal, perinatal, and post discharge care) (17).

Despite our findings may not apply to all Italian NICUs, this study allows for gaining further insight into the organizational policies within the neonatal intensive care setting. Moreover, it addresses the operational barriers in supporting breastfeeding in preterm infants. The main limitation of the present study is not having collected the breastfeeding rates in the enrolled NICUs. The availability of these data could have been useful for exploring the correlation between the organizational policies and breastfeeding rates at discharge. Furthermore, data were reported only on the basis of what the head nurses stated without performing a qualitative analysis of the breastfeeding policies available in each NICU.

## Conclusions

Based on the present findings, enrolled NICUs appear to provide breastfeeding-supportive environments with special regard to breastfeeding policies, milk expression practices, interprofessional collaboration, and continuity of care. Health care professionals should exert efforts to ensure continuous and updated breastfeeding staff education and promote parent-infant closeness and family centered care.

## Data Availability

The dataset available upon request to the corresponding author.

## Ethics Statement

The Ethics Committee of the Fondazione Istituto di Ricovero e Cura a Carattere Scientifico Cà Granda Ospedale Maggiore Policlinico approved the study and written informed consent was obtained by all participants.

## Author Contributions

ET and MG conceived and designed the study and wrote the article. EB, GS, and LP collected the data and were responsible for database management. PS and DM performed the analysis and contributed to the discussion of the results and FM provided suggestions concerning the content and concept of the article and was responsible for critically revising the manuscript.

### Conflict of Interest Statement

The authors declare that the research was conducted in the absence of any commercial or financial relationships that could be construed as a potential conflict of interest.
